# A tool for the interactive analysis and exploration of in-vivo haemodynamics from 4D PC MRI

**DOI:** 10.1186/1532-429X-14-S1-W70

**Published:** 2012-02-01

**Authors:** Johann B Drexl, Anja Hennemuth, Sebastian Meier, Ramona Lorenz, Jelena Bock, Andreas Harloff, Michael Markl, Horst K Hahn

**Affiliations:** 1 Fraunhofer MEVIS, Bremen, Germany; 2 Department of Radiology, Medical Physics, University Medical Center Freiburg, Freiburg, Germany; 3 Departments of Radiology and Biomedical Engineering, Northwestern University Feinberg School of Medicine, Chicago, IL, USA; 4 Neurology, Universitätsklinik Freiburg, Freiburg, Germany

## Background

Time-resolved 3D (4D) phase contrast (PC) MRI allows deriving anatomical as well as functional information in the cardiovascular system. Progress in 4D MR techniques now facilitates volumetric, 3-directional, cine PC MRI data in reasonable scan times. The analysis of these data is, however, a challenging task because of the data complexity (3 spatial dimensions, 3 velocity directions, time) and the many processing steps required. Current evaluations of 4D PC MRI data often use a combination of home built and commercial tools, which are tailored to often time consuming (up to several hours) workflows for special research questions. The purpose of this study was to develop and evaluate a novel analysis tool that allows a fast interactive exploration of patient-specific 4D-PC hemodynamics.

## Methods

**Figure 1:**
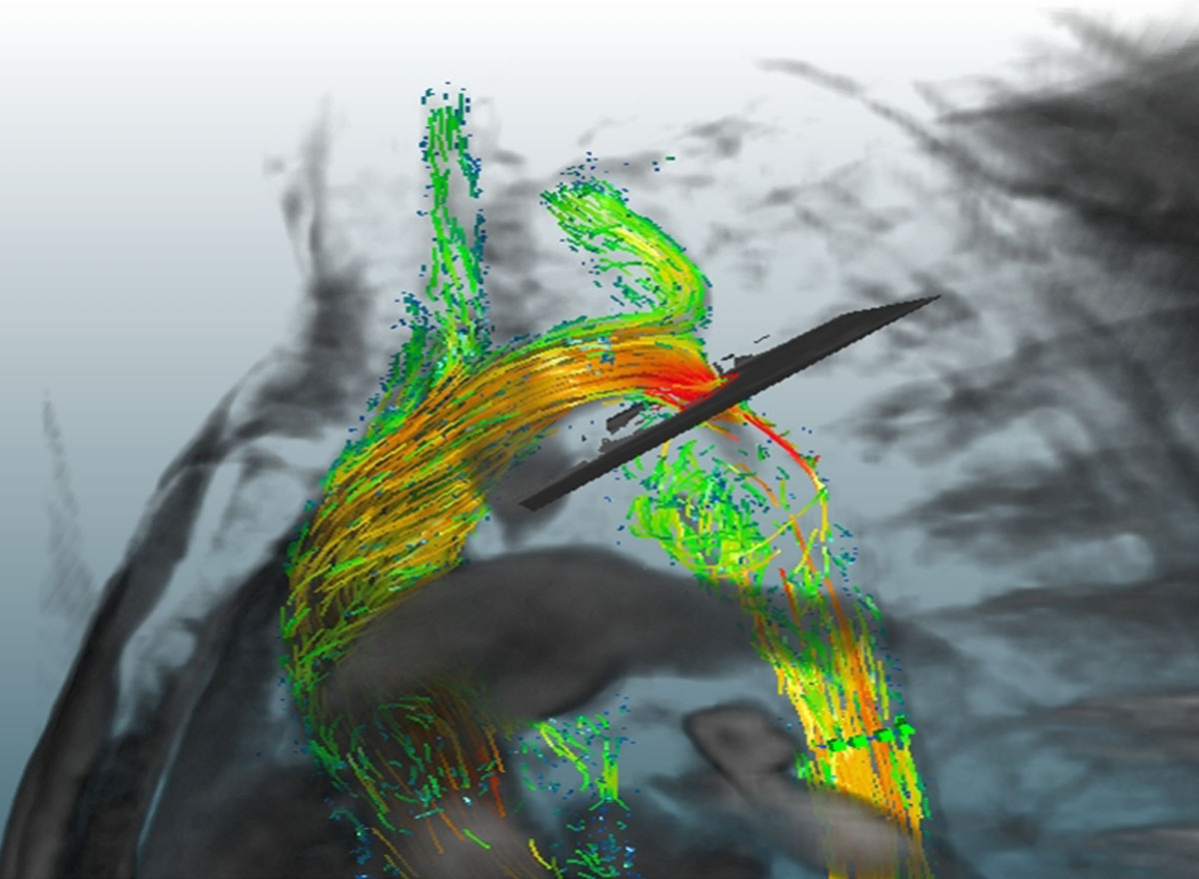
Visualization of flow pathways

The tool provides a combination of pre-processing and analysis methods including 4D phase offset error correction with 3rd order polynomials, antialiasing to correct of phase wraps, semi-automatic vessel segmentation, 3D flow visualization, and calculations of 4D pressure difference maps within the vessel volume. Based on this information, mean velocities and flow rates are calculated at user-defined cross sections of the vessel. Forward as well as backward tracking of pathlines emitted from user defined regions allows the visualization of flow pathways, which can be color-coded according to local velocity, direction, curvature or their emitter region (Fig 1).

## Results

Experts with different clinical specialization analyzed a total of 11 cases (thoracic as well as cranial datasets). The evaluation consisted of the segmentation of the vessels of interest, animated visualizations of flow pathways, placement of several regions of interest, inspection and export of quantitative measurements of velocity and flow. The average processing times were 35.3min +/-14.4 min for the thoracic datasets and 45.2min+/-11.1 min for the cranial datasets.

In addition, our clinical partners provided us with Doppler ultrasound measurements for 5 additional aortical cases with aortic coarctation, which we utilized for an assessment of the velocity estimation functionality in our tool. The 4D PCMRI datasets of these patients were analyzed using our tool, and measurements of peak velocity were performed at cross sections placed in the coarctation.

**Figure 2:**
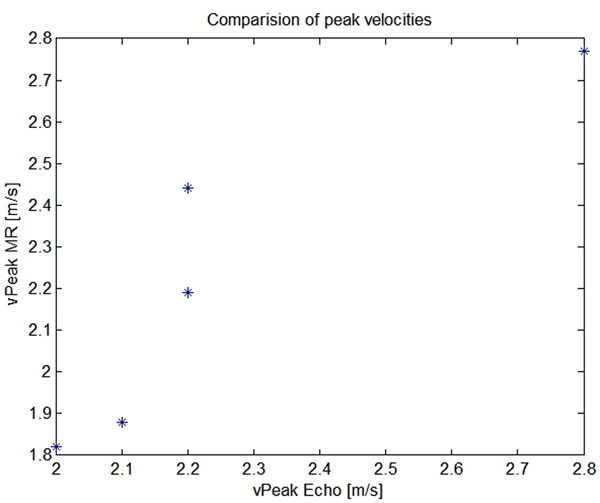
Comparision of peak velocities measured by Doppler and by MRI

We found a correlation coefficient of r=0.90 between the peak velocities measured by Doppler and the peak velocities computed by our tool from PC MRI data(Fig 2).

## Conclusions

We presented a software tool that provides the improved functionality for the analysis and exploration of 4D PC MRI data including pre-processing, segmentation, quantification and visual exploration of flow patterns. We demonstrated the usability of our tool by clinical experts, and we presented a comparison with the clinically accepted Doppler ultrasound method.

## Funding

Funded by the Deutsche Forschungsgemeinschaft project "Plaques der Aorta als Emboliequelle für Schlaganfälle" FR 2795/2-1.

